# Microbiota of table olive fermentations and criteria of selection for their use as starters

**DOI:** 10.3389/fmicb.2013.00143

**Published:** 2013-06-12

**Authors:** Dilek Heperkan

**Affiliations:** Department of Food Engineering, Faculty of Chemical and Metallurgical Engineering, Istanbul Technical UniversityIstanbul, Turkey

**Keywords:** olive starters, microbiota, Lactobacillus, classification, starter selection

## Abstract

Fermentation is one of the oldest methods for preserving of olives applied worldwide for thousands of years. However, olive processing is a speculative area where whether olives are fermented products or pickled products produced by organic acids and salt. Although lactobacilli and yeasts play a major role in the process, literature survey indicates that lactobacilli are less relevant at least in some types of natural green olives during fermentation. There have been significant advances recently in understanding the process to produce olives, especially the role of lactic acid bacteria and yeasts including biofilm formation on olive surfaces by these organisms. The purpose of this paper is to review the latest developments regarding the microbiota of olives on the basis of olive types, their role on the fermentation process, the interaction between both group of microorganisms and the olive surface, the possibility to use starter cultures and the criteria to select appropriate cultures.

## Introduction

The addition of a starter culture in the production of fermented olives is not a common practice worldwide. Fermentation is usually carried out by the indigenous microbiota. During storage, the top of the storage vessel gets covered with a thick mold layer and mycotoxin such as citrinin formation is observed in olives occasionally (Heperkan et al., 2009). Although, there are no reports published on food intoxication of fungal origin there are reports published on *Clostridium botulinum* type B and its neurotoxin detection in the conserved olives (Jalava et al., 2011). It is not usual, but there are several cases of food-borne botulism described linked to consumption of conserved olives (Cawthorne et al., 2005; Jalava et al., 2011). Spontaneous fermentations have many disadvantages compared to fermentations with starter cultures. The structural and sensory characteristics of a product are improved by using starter cultures and the growth risk of harmful organisms can be prevented. Therefore, interest in the development and use of starter cultures for table olive fermentation is increasing in order to achieve a more controlled process (Panagou et al., 2008; Randazzo et al., 2010; Corsetti et al., 2012).

Table olives are important products of the cultivated olive tree (*Olea europaea* L.). Table olive producing regions extend from the Mediterranean Basin to America, Australia and the Middle East. The world production of table olives exceed 2,000,000 tonnes (2,565,000 tonnes in 2011/2012) per year (International Olive Oil Council, IOOC, 2004) and 50% of the total production is produced by the leading countries Spain, Italy, Greece and Turkey. Table olives are produced from specifically cultivated fruit varieties harvested at the pre-determined stage of maturation (Randazzo et al., 2012). Each olive-growing country has its own typical olive varieties and the production methods vary according to local tradition (Ercolini et al., 2006; Rejano et al., 2010).

## Classification of table olives

**Figure 1:**
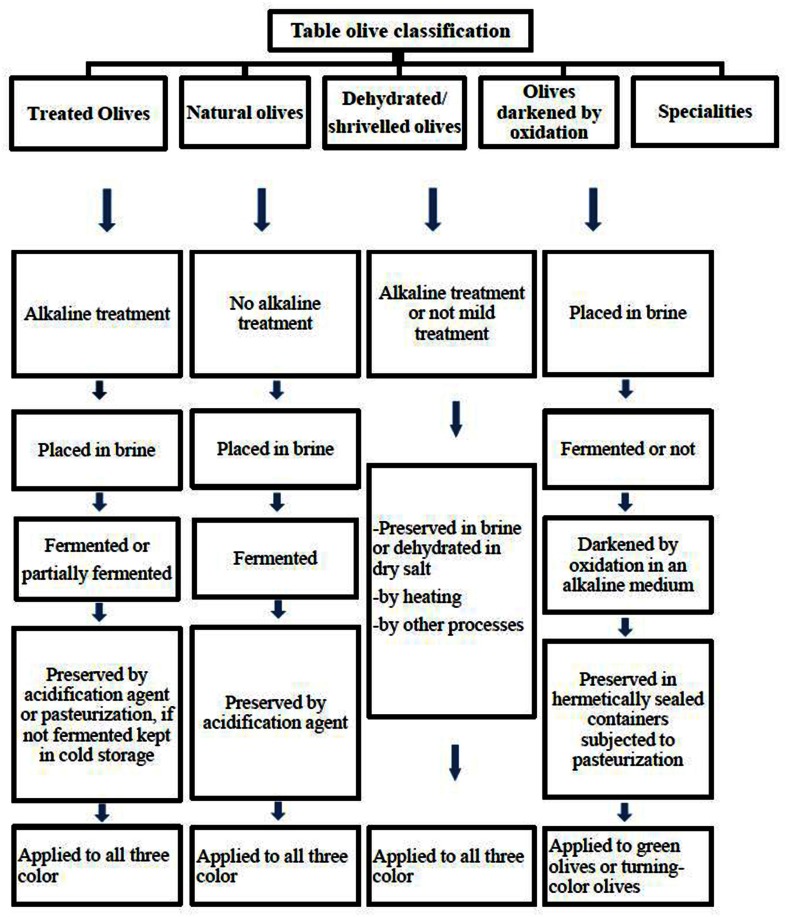
**Classifications of olives based on trade preparations**.

Table olives are classified depending on the processing method by the International Olive Oil Council (IOOC, 2004). Figure 1 reports an example of different commercial preparation of table olives. Olives are inedible due to bitterness, and has to be treated for further consumption (Garcia et al., 2004). The bitter compound oleuropein is a phenolic compound and hydrolyzed by treatment of raw olives with alkaline. Sodium hydroxide (1.8–2.5%, w/v) is used for the treatment followed by a washing step to remove the excess alkali in Spanish-style green olives (De Castro et al., 2002; Aponte et al., 2012). The debittered fruits are then covered with brine (8–10%NaCl) and fermented spontaneously (Domínguez-Manzano et al., 2012). There is no debittering process with NaOH solution in natural olives (Figure 1). Only treated and natural olives have to be fermented and thus conserved by lactic acid bacteria (IOOC, 2004); thus, the characteristics of the olive are preserved and the final product improved (Sánchez-Gómez et al., 2006; Hurtado et al., 2012).

## The microbiota of olives

Table olive fermentations occur spontaneously in many cases without adding any starter culture. The microbiota of olives vary somewhat from cultivar to cultivar and the type of olive processing. The microbiota of olives during fermentation is shown in Table 1. The microbiota of processed olives or brines of processed olives include members of Enterobacteriaceae, *Clostridium*, *Pseudomonas*, *Staphylococcus*, lactic acid bacteria (LAB), yeasts, and occasionally moulds.

**Table 1:**
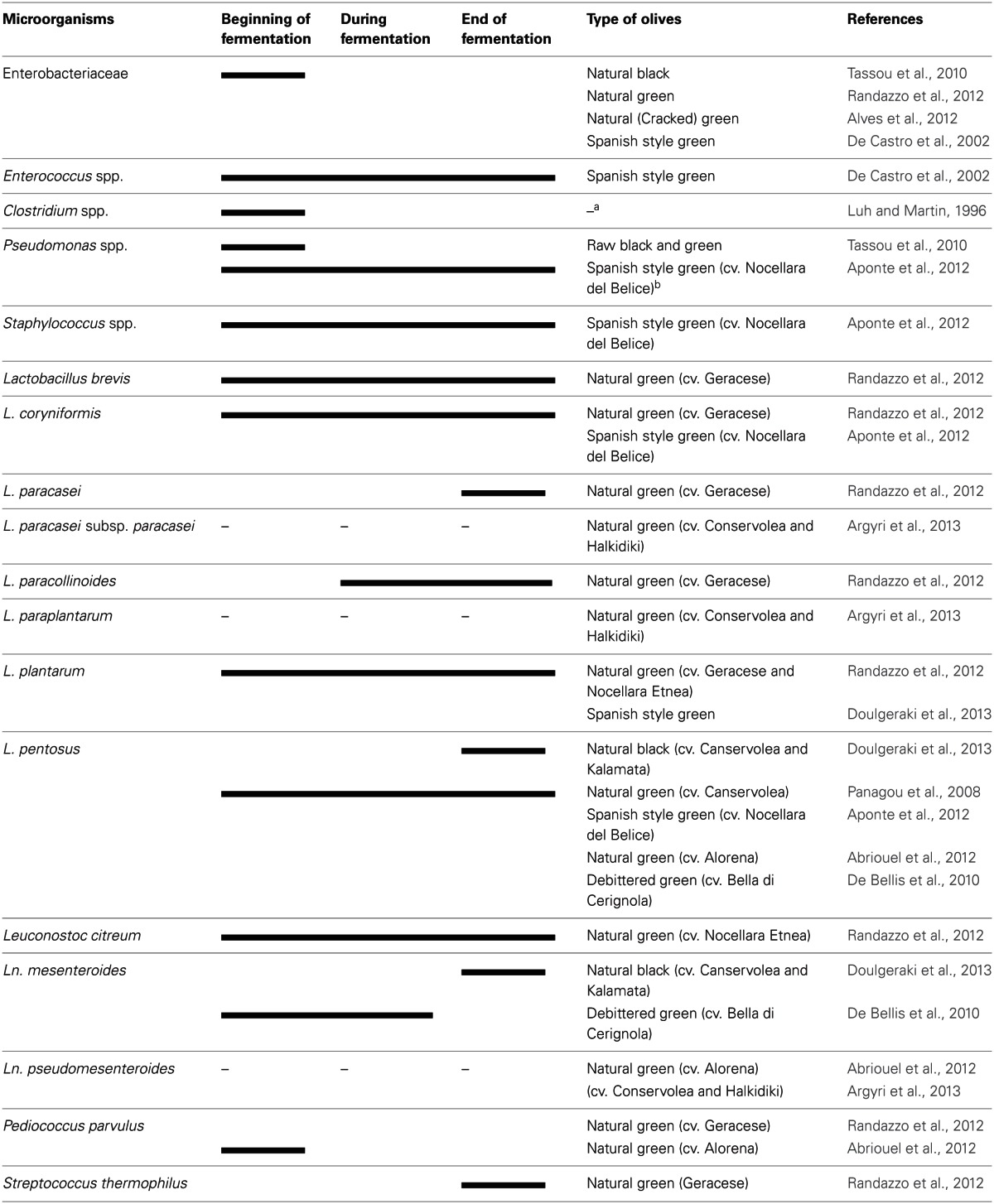
**Microbiota during olive fermentation**.

^a^No information.

^b^cv., cultivar.

The growth of Enterobacteriaceae members were observed in olives at the beginning of fermentation (De Castro et al., 2002; Tassou et al., 2010; Alves et al., 2012; Randazzo et al., 2012). These groups were completely eliminated during fermentation and not detected at the end of the process. Enterobacteriaceae levels ranged from 2.6 to 3.5 log CFU/mL in the brine obtained from cracked green table olives, not debittered with the lye solution, but no viable counts (<10 CFU/mL) were found at the end of fermentation (Alves et al., 2012). *Clostridium* and *Pseudomonas* species can be found at the beginning of fermentation, however, *Clostridium* was unable to survive till the end of the process due to the low pH. The maximum pH value is established by IOOC at 4.1 when olive preserved by its own physicochemical characteristics, or 4.3 when it is preserved by pasteurization (Montano et al., 2010). *Clostridium botulinum* was isolated from only heat treated /conserved olives (Pereira et al., 2008) where the pH of the jar was above 4.6 (Cawthorne et al., 2005). Other possible reasons may be defects associated with the processing, packaging and transportation of the implicated product since several jars had cap leakage and their content was spoiled (Jalava et al., 2011). *Pseudomonas savastanoi* caused the endemic disease, olive knot or tubercle and was isolated from raw olives (Tassou et al., 2010). Therefore, both *Pseudomonas* and *Clostridium* may not be a problem in fermented olives under suitable conditions.

## The role of lactic acid bacteria and yeasts in table olive fermentations

Lactic acid bacteria, which convert fermentable sugars to lactic acid and other organic acids depending on their metabolic pathways, are the most important group of bacteria in olives. Homo fermentative LAB such as *Lactobacillus*, *Streptococcus* and *Pediococcus* and hetero fermentative LAB such as *Leuconostoc* and some members of *Lactobacillus* were detected in fermented olives (Abriouel et al., 2012; Randazzo et al., 2012). However, *Lactobacillus* spp. plays a major role in the process and *Leuconostoc* and *Pediococcus* to a lesser extent (Abriouel et al., 2011; Corsetti et al., 2012). On the contrary, LAB were not detected in some types of natural green olives (Valencic et al., 2010; Alves et al., 2012; Aponte et al., 2012). The changes in the LAB population in Spanish-style green table olive fermentations, studied in detail by Bautista-Gallego et al. (2013) has been published recently. LAB was not detected only in one type (cv.Alorena), directly brined olives stored in a cold room, during the period of study. However, LAB was detected in the other two types of treated olives (cv.Gordal and Manzanilla) (Bautista-Gallego et al., 2013). *Lactobacillus pentosus* was the predominant species in their study (96.4%) whereas *L. plantarum* was rare. *Lactobacillus pentosus* was also the predominant species (81.9%), followed by *Leuconostoc pseudomesenteroides* (10.4%) and *Pediococcus parvulus* (7.6%) in samples isolated from natural green (cv. Alorena) olives during 6 months of fermentation in Spain (Abriouel et al., 2012). In addition to *L. pentosus, Leuconostoc mesenteroides* was also the dominant species in natural black olives (cv.Conservolea and Kalamata) of Greek origin (Doulgeraki et al., 2013). However, *L. plantarum* was determined mainly in Spanish-style green olives of Greek origin as a dominant species in the same study. *Lactobacillus paraplantarum* and *Leuconostoc pseudomesenteroides* were two other species found rarely in natural green and natural black olives, respectively (Bautista-Gallego et al., 2013; Doulgeraki et al., 2013). Two olive cultivars namely Nocellara Etnea and Geracese (natural green olives) were investigated for their microbiota in Italy (Randazzo et al., 2012). Geracese cultivar exhibited wide biodiversity within LAB population in samples fermented under laboratory conditions. The olives were inoculated with *L. plantarum* and *L. casei* and kept for 180 days at room tempeature. *Lactobacillus brevis, L. coryniformis, L. plantarum* and *Leuconostoc citreum* adapted well to brine conditions and revealed throughout fermentation (Table 1). On the other hand, *L. paracollinoides, L. paracasei* and *Streptococcus thermophilus* were detected during and at the end of the process, respectively.

Together with LAB, yeasts play a substantial role in fermented olive production. Since LAB are partially inhibited in directly brined green and natural black olives due to the presence of phenolic compounds, yeasts become especially important (Arroyo-López et al., 2012a). Fermentative yeasts can contribute to the organoleptic characteristics of table olives (Aponte et al., 2012). Yeasts population in olives is shown in Table 2. The most frequently isolated genera are *Candida*, *Pichia*, *Saccharomyces*, and to a lesser extent, *Debaryomyces*, *Issatchenkia*, *Zygotorulaspora*, and *Wickerhamomyces* from different olive varieties (Arroyo López et al., 2006; Coton et al., 2006; Nisiotou et al., 2010; Bautista-Gallego et al., 2011; Alves et al., 2012). The technological properties of yeasts that could be considered in their selection as starters have been reviewed recently by Arroyo-López et al. (2012b). It was reported that among yeasts, *W. anomalus*, *S. cerevisiae*, and *P. membranifaciens* exhibited potential to be used as starters (Bautista-Gallego et al., 2011; Arroyo-López et al., 2012b). Especially *W. anomalus* is well-adapted to the environmental conditions such as low pH and high NaCl concentrations in addition to its other interesting technological properties (Bautista-Gallego et al., 2011). Alves et al. (2012) found that counts of the yeast population increased during fermentation from 4.9 to 5.0 log CFU/mL at the beginning of the process to 6.0–6.5 log 10 CFU/mL at the end of the process. On the other hand oxidative yeasts should be kept at low numbers, since they oxidize lactic acid, raise the pH and thereby may cause spoilage (Aponte et al., 2012). Arroyo-López et al. (2012b) reported that an excessive growth of fermentative yeast species also cause spoilage of olives. Production of high amounts of CO_2_ which results in blister formation reported frequently (Arroyo-López et al., 2012b). Mould genera such as *Aureobasidium, Geotrichum*, and *Penicillium* were also detected in olives to a lesser extent (Table 2). *Penicillium* can grow on the surface of naturally fermented black olives. Mould growth can cause softening of the olive tissue, a mouldy taste and appearance and producing mycotoxins (Heperkan et al., 2006).

**Table 2:** **Yeast and mould species isolated from olives**.

**Yeast and mould species**	**Type of processing/olive**	**Country of origin**	**References**
**YEASTS**			
*Candida apicola*	Cracked directly brined green	Spain	Arroyo López et al., 2006
*C. boidinii*	Ripe black	Spain	Arroyo López et al., 2006
	Directly brined black	France	Coton et al., 2006
	Cracked directly brined green	Portugal	Alves et al., 2012
	Directly brined green	Spain	Bautista-Gallego et al., 2011
*C. diddensiae*	Cracked directly brined green	Spain	Abriouel et al., 2011
		Portugal	Alves et al., 2012
	Directly brined green	Spain	Bautista-Gallego et al., 2011
*C. oleophila*	Cracked directly brined green	Portugal	Alves et al., 2012
*C. olivae*	Directly brined black	Greece	Nisiotou et al., 2010
*C. parapsilosis*	Directly brined green	Italy	Aponte et al., 2010
*C. quercitrusa*	Cracked directly brined green	Portugal	Alves et al., 2012
*C. sorbosa*	Directly brined green	Spain	Hurtado et al., 2008
*C. tropicalis*	Directly brined green Spanish style green	Spain	Bautista-Gallego et al., 2011
*Citeromyces matritensis*	Cracked directly brined green	Portugal	Alves et al., 2012
*Debaryomyces etchelsii*	Directly brined black	France	Coton et al., 2006
	Spanish style green	Spain	Bautista-Gallego et al., 2011
*D. hansenii*	Directly brined black	Greece	Nisiotou et al., 2010
*Issatchenkia occidentalis*	Cracked directly brined green	Spain	Arroyo López et al., 2006
*Pichia galeiformis*	Ripe black	Spain	Arroyo López et al., 2006; Rodríguez-Gómez et al., 2010
	Cracked directly brined green		Abriouel et al., 2011
	Directly brined green		Bautista-Gallego et al., 2011
*P. guillermondii*	Directly brined green	Italy	Aponte et al., 2010
*P. kluyveri*	Directly brined green	Italy	Aponte et al., 2010
*P. membranifaciens*	Directly brined black	Greece	Nisiotou et al., 2010
	Directly brined green	Spain	Bautista-Gallego et al., 2011
*Rhodotorula mucilaginosa*	Cracked directly brined green	Portugal	Alves et al., 2012
*Saccharomyces cerevisiae*	Cracked directly brined green	Spain	Arroyo López et al., 2006
		Portugal	Alves et al., 2012
	Directly brined green	Spain	Bautista-Gallego et al., 2011
*Zygotorulaspora mrakii*	Cracked directly brined green	Portugal	Alves et al., 2012
*Wickerhamomyces anomalus*	Directly brined black	France	Coton et al., 2006
		Greece	Nisiotou et al., 2010
	Directly brined green	Spain	Bautista-Gallego et al., 2011
**MOULDS**			
*Aureobasidium pullulans*	Cracked directly brined green	Portugal	Alves et al., 2012
	Directly brined black	Greece	Nisiotou et al., 2010
*Geotrichum candidum*	Cracked directly brined green	Spain	Arroyo López et al., 2006
*Penicillium citrinum*	Natural fermented black	Turkey	Heperkan et al., 2006
*P. roqueforti*	Natural fermented black	Turkey	Heperkan et al., 2006
*P. brevicompactum*	Natural fermented black	Turkey	Heperkan et al., 2006

Recently, the olive epidermis processed according to Spanish-style was examined during fermentation and it was found that LAB such as *L. pentosus*, yeasts such as *Pichia galeiformis* and *Candida sorbosa* and moulds such as *Geotrichum candidum* coexisted in the microflora of olives (Arroyo-López et al., 2012c). The authors also reported that LAB and yeasts colonized the olive epidermis starting at the 10th day of fermentation till the 3rd month which was the end of the process.

## Microorganisms used as starter cultures in table olive fermentations

Fermentation is a process dependent on the biological activity of microorganisms for the production of a range of metabolites (Ross et al., 2002). Table olive fermentations occur spontaneously in many cases without adding any starter culture (Ruiz-Barba and Jiménez-Díaz, 2012). However, interest in the development and use of starter cultures for table olive production is increasing (Randazzo et al., 2012). Actually there has been several attempts to develop single or two strain starter cultures at a pilot-plant scale (Aponte et al., 2012; Domínguez-Manzano et al., 2012). More recently it was reported that a new starter culture consisting of two *L. pentosus* strains was developed and used for the production in Spanish-style green olives (Ruiz-Barba and Jiménez-Díaz, 2012). The authors explained that this starter culture has been used extensively in the industry in Spain, other Mediterranean countries and Argentina.

Starter cultures are preparations of live microorganisms or their resting forms, whose metabolic activity has desired effects in the fermentation substrate (Bevilacqua et al., 2012). Addition of a starter culture improves the process and contributes to more control over aroma, texture and flavor of the final product (Holzapfel, 2002; Leroy and De Vuyst, 2004; Porto-Fett et al., 2008; Aponte et al., 2012).

LAB and yeasts play an important role in the production of treated and natural table olives (Arroyo-López et al., 2012c). *Lactobacillus plantarum* and *L. pentosus* seem to be the most relevant LAB species as starter culture for natural black, natural and treated olives, respectively (Table 1). *Lactobacillus* spp. coexists in the cover brine with a diverse yeast population during the fermentation process (Garrido Fernandez et al., 1997; Domínguez-Manzano et al., 2012). Some interactions between yeasts and LAB in table olive fermentations have already been described (Nychas et al., 2002; Arroyo-López et al., 2008, 2012c; Domínguez-Manzano et al., 2012). *Lactobacillus pentosus* and *Saccharomyces cerevisiae* is a good example of positive interaction in green table olives (Segovia-Bravo et al., 2007). Additionally it was reported that, *Candida diddensiae* and *L. pentosus* co-inoculated together led to a better microbial development profile than single inoculations (Hurtado et al., 2011). *Lactobacillus pentosus* and yeast populations were able to form mixed biofilms (starting from the 7th day of the process) throughout the fermentation process on both glass slides and the olive skin (Domínguez-Manzano et al., 2012). The authors also found that, olives and brines are similar sources of LAB, however, the former is being higher source of yeast. *G. candidum*, *P. galeiformis*, and *C. sorbosa* were the main yeast species isolated from mixed biofilms containing *L. pentosus* in another study (Arroyo-López et al., 2012c). *Candida boidinii* was also shown to have a capacity of adhesion and colonization on the olive skin, thereby proving to be a possible starter culture (Arroyo-López et al., 2012c). *Enterococcus* spp. such as *E. casseliflavus* as well as other species was studied as a starter culture for Spanish-style green olives together with *Lactobacillus* (De Castro et al., 2002). On the other hand, enterococci which can cause infections in humans have not been recommended by the European Food Safety Authority (EFSA, 2007).

## Criteria for selection of strains as starter culture

Microorganisms (species) to be used as starter culture vary considerably depending on the type of fermented product. However, strains selected as starter culture should have some common characteristics which are explained below.

Factors determining the selection of starter cultures include suitability for their phenotypic characteristics and technological properties. The cultures should improve nutritional properties and improve health aspects of the product and develop better flavor/aroma as well (Corsetti et al., 2012). Lactic acid bacteria in some fermented foods may be divided into two groups such as starter and non-starter lactic acid bacteria (NSLABs) based on their role during the fermentation process. NSLABs can play an important role in ripening and flavor development in fermented foods such as cheese (O'Sullivan et al., 2013), whereas starter lactic acid bacteria (SLAB) ferment lactose and produce high concentrations of lactic acid (Settanni and Moschetti, 2010). There are of course differences between the population numbers of SLAB and NSLAB throughout the process. The former are high in number (≥10^8^ cfu/g) at the beginning of fermentation, may decrease during fermentation and most of them may not even be present in the final product. However, this differentiation for LAB related to olive production may not be as distinct as in cheese. The SLAB strain used as starter culture in olives may have some of the advantages of NSLAB in cheese such as preservation of foods by avoiding growth of both pathogenic and spoilage microorganisms and health benefits such as probiotic characteristics. Several related studies exist in literature; among reported strains used as a starter culture in olives, *L. pentosus*, *L. plantarum*, and *L. paracasei* are potential probiotic bacteria (Nguyen et al., 2007; De Bellis et al., 2010; Argyri et al., 2013). In addition, these three species have more or less some antimicrobial potential. *Lactobacillus pentosus* (Todorov and Dicks, 2007) and *L. plantarum* (Van Reenen et al., 2003) inhibit a number of gram positive as well as gram negative bacteria, whereas *L. paracasei* has a very narrow antimicrobial spectrum, limited to several strains that belongs to closely related species (Tolinacki et al., 2010).

The survival of strains upon freeze drying is crucial. Since this is strain dependent, a strain's survival during freeze drying and storage must be studied before further development as a starter culture (Edward et al., 2011). The growth rate of a particular strain is another important aspect. The delay at the onset of fermentation may expose the production to a high risk of spoilage (Aponte et al., 2012). In addition to phenotypic characteristic of a particular strain, technological characteristics like survival in brine, production of high amounts of lactic acid during fermentation, tolerance to high pH values or adhesion to olive surface are other factors important for final selection (Hurtado et al., 2012; Ruiz-Barba and Jiménez-Díaz, 2012; Bautista-Gallego et al., 2013). The ability to grow at a pH above 9–10 will provide the starter culture with an additional selective advantage over the natural microbiota. Since the olives are treated with NaOH, pH values at the beginning of fermentation are usually high in Spanish-style green olives (Ruiz-Barba and Jiménez-Díaz, 2012). The number of bacteria is also important and thus Dellaglio et al. (2005) suggested that in order for bacteria to exert their beneficial effects, it is necessary that they are present in sufficient numbers.

Certain Lactobacillus species such as *L. pentosus* are able to colonize olive epidermis (Domínguez-Manzano et al., 2012). This strain initially inoculated in the cover brine and to predominate during fermentation (Arroyo-López et al., 2012c). Yeast species were isolated from these biofilms as well (Domínguez-Manzano et al., 2012). Thus starter culturs play an important role to provide a base to adhere for other useful microorganisms. Certain yeast species such as *Candida boidinii* have the capacity of colonization and formation of biofilm on olive skin (Arroyo-López et al., 2012c).

## Methods used for selection of appropriate strains as starter culture

Since LAB and yeast play an important role in fermented olive production, microorganisms to be used as starter culture are preferably selected from LAB and yeast species already present in the olive microbiota. Many different approaches are used for the determination of microbial population of olives and identification of species that have been isolated. In recent studies, using culture-dependent methods, LAB or yeast cultures were isolated from olives and/or brine by using selective media such as de Man, Rogosa and Sharp (MRS) agar (Oxoid) and oxytetracycline-glucose-yeast extract (OGYE) agar (Oxoid), respectively. In this method, each isolate is checked for purity and then subjected to identification. Polyphasic approaches as suggested by a number of researchers are quite useful in the identification of a particular strain. For this purpose both phenotypic and genotypic characteristics can be used. Molecular techniques such as PCR is quite useful in comparing the genetic similarity between organisms. Individual genes or whole genomes can be used in sequence analyses. In the identification of LAB, 16S rRNA gene is employed. In the case of one genome sequence, micro-arrays can be designed (Dellaglio et al., 2005). Botta and Cocolin (2012) suggest, however, that a comparison between the results of culture-dependent and -independent studies for the determination and eventually selection of the main species of LAB and yeasts involved in olive fermentation is preferable.

More recently, methods called culture-independent, in which DNA or RNA are extracted directly and analyzed from the food matrix (Botta and Cocolin, 2012) are employed in various products including olives. In this system, all organisms, including those that cannot be cultured are detected (Dellaglio et al., 2005). Denaturing gradient gel electrophoresis (DGGE), fluorescence *in situ* hybridization (FISH) and multiplex PCR have been used for this purpose. DGGE is able to follow dynamic changes that occur during food fermentation and highlight dominant microbial populations (Cocolin et al., 2007). This technique (PCR-DGGE) allows to assess the microbial diversity as well as the metabolic potential of the microbial communities in any ecosystem (Leite et al., 2012). PCR-DGGE was used for the determination of the diversity of bacteria, archaea, yeasts and molds in different olive fermentations (Abriouel et al., 2011). DGGE is a useful tool for the assessment of microbial diversity and follow the behavior of starter cultures during olive fermentation. Fluorescence *in situ* hybridization (FISH) with 16S rRNA probes was another technique used to assess the members of the lactobacilli in fermented olives (Ercolini et al., 2006; Hurtado et al., 2012). FISH is very useful to detect microorganisms directly in their habitats without culture dependent isolation of cells or culture independent extraction of nucleic acids prior to the identification (Ercolini et al., 2006). However, when data obtained from FISH were insufficient to resolve the species level of the isolates (especially discriminating *L. plantarum* group), specific multiplex PCR assays targeting the *rec*A or *tuf* genes were employed (Torriani et al., 2001; Doulgeraki et al., 2013). This method applied successfully in olives for discriminating closely related species such as *L. plantarum*, *L. pentosus*, and *L. paraplantarum* (Hurtado et al., 2011; Doulgeraki et al., 2013). Multiplex PCR is the other culture-independent technique which allows the screening and characterization of lactic acid bacteria and yeasts in fermented olive during processing and storage (Doulgeraki et al., 2012; Bautista-Gallego et al., 2013).

For yeast identification, several PCR-based methods are developed. In olives, the identification of yeast strains was performed by the PCR-RFLP method described by Esteve-Zarzoso et al. (1999) in combination with a sequence analysis of the D1/D2 domain of 26S rDNA. For PCR amplification, ITS 5.8 rRNA region was used except for the primers (NL1 and NL4) (Alves et al., 2012). These methods used for identification appear to be adequate to characterize the yeast biota. However, there are other methods reported which are not yet applied to olives. Matrix-assisted laser desorption ionization time of flight mass spectrometry (MALDI-TOF MS) has been employed recently for the identification and differentiation of yeast isolates including closely related *Candida* spp. (Dhiman et al., 2011). This was shown to be a rapid and reliable tool for the accurate identification of *Candida* isolates directly from colonies within minutes, more suitable for routine analyses in a medical laboratory (Ferroni et al., 2010; Goyer et al., 2012). Although the equipment is expensive, the low cost of its consumables, the extreme speed, and elimination of false negative results are some of the advantages of the instrumentation (Vranakis et al., 2012). The method can be adapted to LAB and yeast identification in olives. Microbiological analyses for olives may become routine tests in the future.

Selection of appropriate cultures is a complex process as explained in detail above. Once selected, validation on a lab-scale basis and validation at the factory-scale are recommended (Bevilacqua et al., 2012). These steps are also critical and affect the safety and quality of the final product.

## Probiotic characteristics of starter cultures

Probiotic potential is another significant character of a LAB for the selection of certain strains as potential starter cultures (Doulgeraki et al., 2013). The most commonly used LAB species, in probiotic preparations are *Lactobacillus* ssp., *Bifidobacterium* ssp., and *Streptococcus* ssp. (Shah, 2007). Probiotic strains have several beneficial properties such as improving intestinal tract health, producing antimicrobial substances, enhancing the immune response, reducing symptoms of lactose intolerance, enhancing the bioavailability of nutrients, and decreasing the prevalence of allergy in susceptible individuals (Parvez et al., 2006; De Bellis et al., 2010; Mena and Aryana, 2012). The interactions between probiotic strains and traditional starter cultures are another aspect that must be considered (Tamime et al., 2005). Some probiotic microorganisms may influence the organoleptic properties of the fermented products or probiotic microorganisms may, particularly, be adversely influenced by starter culture bacteria (Tamime et al., 2005). Despite some of the interaction, olives may serve as a suitable vehicle to carry probiotics. In recent studies, potentially probiotic cultures of *Lactobacillus* were tested for table olive fermentation and promising results were obtained. For example, the probiotic *L. paracasei* strain (IMPC2.1) successfully colonized both the olive surface (De Bellis et al., 2010) and human gut (Lavermicocca et al., 2005), and eventually dominated the native LAB populations in 30 days (Valerio et al., 2011). Properties of LAB strains, isolated from fermented olives can be investigated to select suitable strains which can be used as probiotic starters instead of bacteria from human and animal sources. With a similar approach Argyri et al. (2013) studied 71 LAB strains from fermented olives and found that more than one strain of *L. pentosus*, *L. plantarum*, and *L. paracasei* subsp. *paracasei* were desirable *in vitro* probiotic properties. Yeast strains selected as starter culture should also be investigated for their probiotic potential. The research efforts in this area are continuing and some promising results were reported for yeasts species isolated from olives such as *C. boidinii*, *C. oleophila*, *D. hansenii*, and *P. membranifaciens* to have a potential (Psani and Kotzekidou, 2006; Silva et al., 2011; Arroyo-López et al., 2012a). The main criteria for probiotic selection documented by FAO/WHO, (2002, 2006) is being used as a guideline by a number of researchers to evaluate the probiotic potential of bacteria and yeasts (Morrow et al., 2012; Peres et al., 2012; Strahinic et al., 2012; Argyri et al., 2013). These criteria include; the survival of bacterial cells during their passage through the gastrointestinal tract and the ability of a strain to colonize transiently mucosa (Guarner et al., 2005; Argyri et al., 2013). The selected probiotic strain may be added to the brine at the initiation of fermentation where it acts as a starter to ensure proper fermentation outcomes (Peres et al., 2012).

## Conclusion

Table olive is a fermented product produced mainly by LAB and yeasts. The microbiota and interaction of bacteria and yeasts are different in natural black and green olives from the lye-treated olives. Table olive fermentations occur spontaneously in many cases, interest in the development and use of starter cultures for table olive production is increasing however. Indeed *L. pentosus, L. paracasei*, and *L. plantarum* have been used as a starter culture in some parts of the world according to reports. The negative and positive aspect of yeast in table olive fermentation has not been fully understood therefore the activity and role of yeasts alone or in combination with LAB in olive fermentation has to be studied in depth. *Candida boidinii* seems to be the most prominent species as a possible starter culture in fermented olives. The microbiota of olives vary somewhat from cultivar to cultivar and the type of olive processing. Starter cultures are thus, an essential part of olive fermentation in order to control the safety and the quality of the end product. Selection of cultures to be used as starters is a complex process that starts from the isolation step followed by strain characterization and determination of their technological properties and eventually validation at factory-scale. Probiotic potential of the selected strain should also be considered. Production of a novel functional food having advantages of probiotic bacteria can add more value to table olives which already have great nutritional value.

### Conflict of interest statement

The author declares that the research was conducted in the absence of any commercial or financial relationships that could be construed as a potential conflict of interest.
